# Adjustment for Baseline Covariates to Increase Efficiency in RCTs with Binary Endpoint: A Comparison of Bayesian and Frequentist Approaches

**DOI:** 10.3390/ijerph18157758

**Published:** 2021-07-22

**Authors:** Paola Berchialla, Veronica Sciannameo, Sara Urru, Corrado Lanera, Danila Azzolina, Dario Gregori, Ileana Baldi

**Affiliations:** 1 Department of Clinical and Biological Sciences, University of Torino, 10100 Torino, Italy; sara.urru@unito.it; 2 Unit of Biostatistics, Epidemiology and Public Health, Department of Cardiac, Thoracic, Vascular Sciences and Public Health, University of Padova, 35121 Padova, Italy; veronica.sciannameo@unito.it (V.S.); corrado.lanera@unipd.it (C.L.); dario.gregori@unipd.it (D.G.); ileana.baldi@unipd.it (I.B.); 3 Department of Medical Sciences, University of Ferrara, 44121 Ferrara, Italy; danila.azzolina@unife.it

**Keywords:** randomized controlled trial, causal inference, doubly robust estimation, propensity score

## Abstract

Background: In a randomized controlled trial (RCT) with binary outcome the estimate of the marginal treatment effect can be biased by prognostic baseline covariates adjustment. Methods that target the marginal odds ratio, allowing for improved precision and power, have been developed. Methods: The performance of different estimators for the treatment effect in the frequentist (targeted maximum likelihood estimator, inverse-probability-of-treatment weighting, parametric G-computation, and the semiparametric locally efficient estimator) and Bayesian (model averaging), adjustment for confounding, and generalized Bayesian causal effect estimation frameworks are assessed and compared in a simulation study under different scenarios. The use of these estimators is illustrated on an RCT in type II diabetes. Results: Model mis-specification does not increase the bias. The approaches that are not doubly robust have increased standard error (SE) under the scenario of mis-specification of the treatment model. The Bayesian estimators showed a higher type II error than frequentist estimators if noisy covariates are included in the treatment model. Conclusions: Adjusting for prognostic baseline covariates in the analysis of RCTs can have more power than intention-to-treat based tests. However, for some classes of model, when the regression model is mis-specified, inflated type I error and potential bias on treatment effect estimate may arise.

## 1. Introduction

Baseline covariates impact the outcome in many randomized controlled trials, and a recent systematic review reported that 84% of the trials present adjusted analysis. Among them, 91% pre-specified in the protocol such adjusted analysis [1]. It has been shown that models that adjust for baseline covariates can substantially improve the statistical power of the analysis when the covariates are moderately to strongly prognostic.

While this is justified for continuous outcomes, for binary outcomes, which require non-linear models, covariate adjustment may change the magnitude of the treatment effect, and thus the situation is subtler [2]. Due to the non-collapsibility of odds ratios, the non-adjusted and adjusted analyses estimate the marginal and the conditional treatment effect, respectively. However, the overall effect of adjusting for baseline covariates in logistic regression is still an increase in power. This is because the marginal estimate is always closer to the null effect than the conditional one, and the impact of the loss of precision on the power of the conditional estimate is offset by the larger effect size, leading to a net increase in power for the adjusted analyses [2,3].

The large amount of baseline covariates collected in an RCT opens the possibility to select the combination of covariates that results in the most favorable treatment effect estimate and/or the lowest *p*-value [4].

This is well-recognized in the “Guideline on adjustment for baseline covariates in clinical trials”, issued by the European Medicines Agency (EMA) in 2015, which requires pre-specification in the protocol of the variables to be included in the primary analysis for preventing the potential selection of the combination of covariates that may influence the treatment effect, especially in non-linear models [5].

However, pre-specification of the variables to be adjusted for is not always feasible as all prognostic variables may be not known in advance.

Under the frequentist approach, doubly robust and semi-parametric efficient estimators allow for the separation of treatment effect estimation from baseline covariate adjustment [6,7,8]. This is achieved by the inverse-probability-of-treatment weighting (IPTW) estimator, the parametric G-computation, the semiparametric locally efficient (SLE) estimator and the more recent targeted maximum likelihood estimator (TMLE). Under the Bayesian framework, model averaging is an alternative to the more common approach of model selection [9], which relies on estimation from a single model. While Bayesian model averaging (BMA) successfully accounts for model uncertainty in making a prediction, its advantages are less straightforward when used within the causal inference framework. In the context of causal treatment effect estimation, BMA tends to assign large posterior probabilities to models that may not accurately adjust for confounding. To overcome this drawback, the Bayesian adjustment for confounding (BAC) algorithm has been proposed as an alternative approach based on the specification of both an outcome and a treatment model, as in the propensity score framework [10].

However, since BMA and BAC are based on models that likely contain noisy prognostic covariates, they lose precision in estimating the treatment effect. To overcome this limitation, the generalized Bayesian causal effect estimation (GBCEE) has been proposed as a further unbiased and efficient estimator [11].

This study investigates which methods of adjusting for baseline covariate in the analysis of RCTs with binary endpoint maximize the statistical power while retaining the type I error rate and unbiased estimate of treatment effect. Such comparison is justified because type I error and power are still the study operating characteristics of concern healthcare regulators require when appraising the results of confirmatory clinical trials [12].

In the following, in Section 2.1, the motivation example is introduced. Then in Section 2.2, the simulation study is explained, and the frequentists and Bayesian estimators are briefly presented. Results of both simulation and the illustrative study will be reported in Section 3 and finally discussed in Section 4.

## 2. Materials and Methods

### 2.1. Illustrative Study and Simulated Data

Our simulation study was based on the motivating example of re-analyzing the PROLOGUE RCT [13]. The PROLOGUE study is among the largest trials investigating whether DPP-4 inhibitors provide cardiovascular protective effects to patients with type 2 diabetes by slowing carotid stiffness progression associated with conventional diabetes treatment.

The study participants were either allocated to add-on DPP-4 inhibitor (sitagliptin) treatment or to continue therapy with conventional anti-diabetic agents. The primary endpoint was the arterial stiffness of annual changes, which resulted in being not significantly different between the two groups. However, the study showed that the decrease in glycated haemoglobin (HbA1c) in patients treated with sitagliptin was superior to conventional therapy, proving a better glycemic control. As a re-analysis of the PROLOGUE study, we then investigated a potential sitagliptin effect on the improvement of HbA1c.

### 2.2. Simulation Study

The simulation study was carried out to compare the performance of several estimators applied to obtain a marginal treatment effect estimate in the case of a binomial outcome and was based on the same scheme adopted in Zhang et al. [14].

There was a 50% chance of being assigned to either the treatment or the control group. The treatment assignment variable (Z) was generated as Bernoulli with P(Z = 1) = P(Z = 0) = 0.5. The assignment Z = 1 corresponds to the treatment group. The baseline covariates were generated as follows:

- $X_{1},X_{3},\;X_{8}$ follow a Normal (0,1) distribution;
- $X_{4}$ follows a Bernoulli (0.3) distribution;
- $X_{6}$ follows a Bernoulli (0.5) distribution;
- $X_{2}$ was generated as $0.2\times X_{1}+0.98\;U_{1}$
- $X_{5}$ was generated as $0.1\times X_{1}+0.2\times X_{2}+0.97\;U_{2}$
- $X_{7}$ was generated as $0.1\times X_{3}+0.99\;U_{3}$

where $U_{1},U_{2},\;U_{3}$ are Normal (0,1) variables.

Finally, Y was generated as Y=logit (P(Y=1|Z, X))=α+βZ+γX, where *X* = *(X*_1_, …, *X*_8_*)* is the matrix of covariates, α=0.9, β=1.3, γ=(0.5, 1.3, 0.5, 1.5,0,0,0,0). The parameter β is the conditional treatment effect; α is the intercept and γ is the vector of the coefficients of covariates $X_{1},\ldots ,X_{8}$. Thus, $X_{1},\ldots ,X_{4}$ variables represent prognostic patient features for treatment effect.

To find the marginal treatment effect, one million individuals were simulated, and 30 repetitions were performed. The marginal treatment effect was then calculated as the mean of the treatment effects as the log odds ratios using unadjusted logistic regression. The true marginal treatment effect resulted in being equal to −0.871 ± 0.004.

For the simulation study, 5000 datasets of sample size *n* = 200 were generated. For the frequentist estimators, several scenarios were defined to evaluate the effects of model selection and are reported in Table 1.

The model estimated under the correct scenario is the same used to generate the outcome data when all prognostic variables are known. The model estimated under the mis-specification scenario includes only one prognostic variable and an additional noisy variable. Finally, the model estimated under the all-variables scenario includes all the prognostic variables as well as non-prognostic variables and mimics the situation of using all patient features for the treatment effect estimation in the case of uncertainty about knowledge on prognostic variables.

#### 2.2.1. Frequentist Estimators

The frequentist estimators employed for the estimation of the treatment effect are briefly presented. In describing the estimators, we will refer to the treatment model as the conditional probability (likelihood) of being treated given the covariates, i.e., P(Z|X), and to the outcome model as the probability, i.e., likelihood, of the outcome given the treatment and the prognostic covariates, i.e., P(Y|Z, X).

*G-computation*. To address confounding, G-computation relies on the estimation of the outcome model, i.e., the conditional expectation of the outcome given the treatment and the prognostic covariates. Contrary to the propensity score methods, it does not require estimating the exposure mechanism or treatment model, i.e., the conditional probability of being treated given the observed confounders [15].

*Doubly Robust Estimation*. Doubly robust (DR) estimation combines the outcome model and treatment model. Both the models are unbiased only if they are correctly specified. The DR estimation ensures that when combining the two models for the treatment effect estimation, only one of them must be correctly specified to obtain an unbiased estimate. The estimates of the parameters of interest of the outcome model and the treatment model are used to predict patients’ responses under the treatment condition and the treatment assignment (propensity score), respectively. Inversely weighting the expected response under treatment condition by the propensity score allows us to represent the estimator of the quantity of interest as an unbiased estimate plus a second term. This term reduces to 0 if either the treatment model or the outcome model are correctly specified and if, and only if, the possibly incorrect conditional density has the same support as the true conditional density [6].

*Semi-Parametric Locally Efficient Estimator*. It uses a semi-parametric model for the outcome model, which is used to generate predicted values separately from the treatment model. Finally, it computes the average treatment effect as the mean difference in predicted outcome pair across individuals [16].

*Targeted Maximum Likelihood Estimator*. TMLE is a doubly robust, maximum-likelihood–based estimation method that includes a secondary targeting step that optimizes the bias-variance tradeoff for the estimation of the parameter of interest. TMLE is particularly attractive for causal effect estimation in RCT analysis. First, it is a doubly robust method, which yields unbiased estimates if the treatment model is correctly specified, which is the case of RCT setup [17].

*Augmented Inverse Probability Weighting*. Propensity scores are estimated and used to create inverse probability weights; all observations are weighted. Finally, it computes the average treatment effect as the mean difference between weighted outcomes among exposed and unexposed [18].

#### 2.2.2. Bayesian Estimators

*Bayesian Model Averaging*. BMA is an extension of the Bayesian inference methods. In addition to the usual modelling of parameter uncertainty through the prior distribution, it models the uncertainty of the model selection process, obtaining a posterior parameter and posterior probability model through Bayes’ theorem. In the present work, we considered Zellner’s g distribution as a-priori distribution on coefficients for the variable selection [19] and the Bayesian adaptive sampling algorithm for the model selection [9].

*Bayesian Adjustment for Confounding*. As in the propensity score framework, BAC requires the definition of the outcome model, which is a function of the treatment and potential confounders, and the treatment model, which is a function of the potential confounders to treatment assignment. Then it applies a Bayesian variable selection process in both models to select covariates and introduces a dependence parameter between the outcome and treatment model, ω, which denotes the prior odds of including a confounder in the outcome model, given that the same confounder is in the exposure model. In the special case of dependence parameter ω = 1, BAC reduces to BMA [10].

*Generalized Bayesian Causal Effect Estimation*. The generalized Bayesian causal effect estimation (GBCEE) algorithm performs variable selection and delivers doubly robust estimates. It employs a prior distribution that targets the selection of true confounders and predictors of the outcome. It thus takes advantage of the Bayesian framework to account for uncertainty in the model selection process. It is different from BMA in building the prior distribution. It uses a prior distribution tailored to identify the potential confounders, which uses information from the data and thus relies on the empirical Bayes approach. Finally, it adds a doubly robust estimation, employing the posterior distribution of the parameters and adopting the TMLE framework to estimate the causal effect that protects against model mis-specification [11].

## 3. Results

### 3.1. Simulation Study

For each method, we computed the bias as the difference between the average of the estimates and the true effect. The standard error (SE) of the estimates, the Monte Carlo standard error for the standard deviation (MC SD) and the coverage probability (CP), i.e., the proportion of simulation replicates in which the 95% confidence intervals included the true effect. For the frequentist estimators, 95% confidence intervals were computed. For the Bayesian estimator GBCEE, 95% confidence intervals were computed as well, using 50 non-parametric bootstrap replicates with the percentile method. For the BAC and BMA approach, the 95% credible intervals were computed.

Type I error and power were also calculated. For both frequentist and Bayesian estimators, type I error was computed simulating 5000 datasets under the null hypothesis that the treatment is not effective. For BAC and BMA, type I error was estimated by the proportion of the simulations incorrectly declared the treatment effective, based on the posterior probability $P\left(\beta \;<\;0|Y,\;X_{1},\ldots ,\;X_{8}\right)\geq 0.95$.

Similarly, the power was calculated as the proportion of simulations that declare the trial successful based on the given decision criteria when the target treatment effect is assumed to be the true value. This approach has been recommended by the FDA [20].

The performance of the frequentist estimators was assessed under three scenarios: the ideal case of the correct model specification (correct scenario); the case of important prognostic variable not identified in the model specification (mis-specification scenario); finally, the case when noisy prognostic variables are introduced in the model (all-variables scenario). In the all-variables scenario, for SLE estimator, a model selection process was foreseen based on backward and forward stepwise techniques.

Bayesian estimators’ performance was assessed on the all-variables scenario only since they do not require selecting a final model but allow for averaging over the space of potential models that could have generated the data.

Results of the simulation study are reported in Table 2. The bias is similar across all methods, while more variation is observed in the power of the estimators, ranging between 84.6% and 94.9%. For Bayesian estimators, the power is given by the posterior probability of observing a treatment marginal effect greater than zero. A slight inflation of type I error is observed, except for BMA, however, it is not greater than 6.5%.

### 3.2. Illustrative Study

To illustrate the effect of baseline adjustment on the treatment effect estimation, we applied the introduced methods to re-analyze the PROLOGUE RCT [13].

The PROLOGUE RCT aimed to investigate whether DPP-4 inhibitors provide cardiovascular protective effects to patients with type 2 diabetes.

The study participants were either allocated to add-on DPP-4 inhibitor (sitagliptin) treatment or to continue therapy with conventional anti-diabetic agents. The study showed that the decrease in glycated haemoglobin (HbA1c) in patients treated with sitagliptin was superior to conventional therapy, proving a better glycemic control.

We set as outcome an improvement of at least 1% in HbA1c, obtaining a dichotomised outcome. This choice is motivated by the observation that two large-scale studies—the UK Prospective Diabetes Study (UKPDS) and the Diabetes Control and Complications Trial (DCCT)—demonstrated that improving HbA1c by 1% (or 11 mmol/mol) for people with type 1 diabetes or type 2 diabetes cuts the risk of microvascular complications by 25%.

As prognostic covariates, we used age (years), gender (female, male), body mass index (BMI, kg/cm^2^), systolic blood pressure (SBP, mmHg), low-density lipoprotein (LDL, mg/dL), high-density lipoprotein (HDL, mg/dL), HbA1c (%), fasting plasma glucose (FPG, mmol/L), dyslipidemia (LDL ≥ 130 mg/dL odds ratio (OR) HDL < 35 mg/dL OR triglyceride ≥ 150 mg/dL OR total cholesterol (=LDL + HDL + (Triglyceride/5)) ≥ 200 mg/dL).

**Figure 1:**
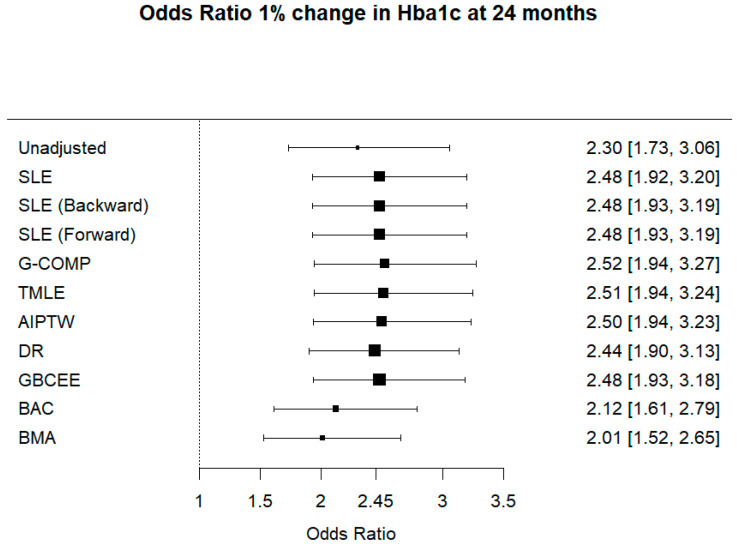
Odds ratio 1% change in Hba1c at 24 months.

In Figure 1, the unadjusted OR of improving HbA1c by 1% is reported along with 95% confidence interval. Frequentist estimates are reported with 95% confidence intervals, and finally, Bayesian estimates with 95% credible intervals are listed.

## 4. Discussion

We have presented a study to compare different approaches to address covariate adjustment to estimate treatment effect in RCTs.

Baseline covariates adjustment impacts the outcome in many RCTs in terms of power, type I error and bias of the marginal effect estimation.

In fact, variable selection methods based on *p*-values, and large observed differences between arms and stepwise approaches, have increased type I error rates [4]. The guideline on adjustment for baseline covariates in clinical trials issued by the EMA in 2015 strongly recommends pre-specifying the variables to be included in the primary analysis in the protocol to avoid bias and potential selection of the combination of covariates that may favour the treatment [4]. Moreover, the Consolidated Standards of Reporting Trials (CONSORT) [21] and the International Conference on Harmonization [22] recommend to pre-specify the potential prognostic variables to employ in adjusted analysis. However, there is still debate on how to identify prognostic covariates correctly.

Several approaches have been proposed to estimate a marginal causal effect, which is the standard measure of treatment effect reported when analyzing RCTs [23]. Although it is known from the literature that adjustment for prognostic covariates can increase the efficiency of the analysis, there is still a lack of attempts to assess comparatively the performance of all the methods under real scenarios of analysis, including adjustment for non-prognostic variables and model mis-specification.

We compared several frequentist and Bayesian estimators under different scenarios. The selected estimators were: SLE estimation, TMLE, G-computation, AIPTW, DR estimator, GBCEE, BMA and the Bayesian adjustment for confounding algorithm. We assessed their performance under three scenarios: the ideal case of the correct model specification; the case of important prognostic variable not identified in the model specification (model mis-specification); finally, the case when noisy prognostic variables are introduced in the model (all variables selected in the adjusted analysis). Since the Bayesian estimators can handle the uncertainty of the model selection process assigning a posterior probability to each set of covariates [24], they were assessed only under the scenario of all variables included in the adjusted analysis.

Our results from the simulation study showed that model mis-specification does not increase the bias. This holds also for the G-computation estimator, which is not theoretically guaranteed to be a consistent estimator under model mis-specification.

The approaches that are not doubly robust have increased MC SD. They also showed increased SE under the scenario of mis-specification.

Across different scenarios, frequentist estimators showed a similar precision (SE ranges between 0.244 and 0.298). This observation is particularly interesting since the correct specification of a parametric model with many covariates is nearly impossible [17]. Bayesian estimators behave differently, showing a high precision for GBCEE (SE = 0.147). The uncertainty of BMA is not directly comparable with SE, since it is the standard deviation computed on the posterior distribution. Thus it showed a larger uncertainty (0.451), which is expected since it embeds the uncertainty of estimates in the posterior probability function.

Covariate adjustment reduced type II error but under the scenario of mis-specification of the outcome model. The Bayesian estimators showed a higher type II error than frequentist estimators under the scenario, including all prognostic variables and noisy covariates in the model specification. On the other hand, BMA showed the largest bias, even if offset by the smallest type I error, which is not surprising since it has been shown that the bias can be relevant when covariates are only slightly associated with the outcome [11].

In the re-analysis of PROLOGUE RCT, we estimated the odds ratio of improving HbA1c using the frequentist and the Bayesian estimators introduced for avoiding confounding. Among the Bayesian estimators, GBCEE resulted similar to other frequentist estimators due to its doubly robust property. In contrast, BMA and BAC showed a smaller treatment effect, compared to the unadjusted estimate.

Adjusting for prognostic covariates leads to an increase in power, as seen by observing that the adjusted estimate is farther from the null value of 1 than the unadjusted estimate (odds ratio equal to 1 indicates no treatment effect). Compared to the unadjusted analysis, we did not observe a dramatic increase of SE, thus a loss of precision, except for BMA and BAC. However, the GBCEE Bayesian estimators showed performances comparable to other frequentist estimators.

## 5. Conclusions

Adjusting for baseline covariates predictive of outcome in the analysis of RCTs can have more power than intention-to-treat based tests. However, for some classes of model, when the regression model is mis-specified, inflated type I error and potential bias on treatment effect estimate may arise. Estimators that allow for separating the baseline covariate adjustment from the treatment effect estimation can avoid potential bias for covariates’ post hoc selection retaining the focus on objective inference on treatment effect. Among Bayesian estimators, BMA presents the largest bias.

Our simulations were carried out in the context of a binary outcome. Similar conclusions are likely to be applied to the hazard ratio since odds-ratio and hazard ratio showed the same non-collapsibility issue.

Limitations of this study rely on the assumption of independent, identical distribution of data, which is not necessarily the case in RCTs. Patients in RCTs often have wide variability in their response to treatment resulting in heterogeneity of treatment effect. Further research should include realistic synthetic datasets, which capture the relationships across clinical features among patients. Probabilistic models, classification-based imputation models, and generative adversarial neural networks are an example of data-driven approaches of synthetic data generation methods [25].

## Figures and Tables

**Table 1:** Scenarios under which the estimators were compared. The model for outcome generation is $Y=\mathrm{logit}\;P(Y|Z,\;X)=0.9+1.3Z+0.5X_{1}+1.3X_{2}+0.5X_{3}+1.5X_{4}$.

Scenario	Outcome Model Estimated	Prognostic Variables in the Outcome Model Estimated	Non-Prognostic Variables in the Outcome Model Estimated
Correct	E(Y|Z, X)=α+βZ+ $\gamma_{1}X_{1}+\gamma_{2}X_{2}+\gamma_{3}X_{3}+\gamma_{4}X_{4}$	$X_{1},X_{2},\;X_{3},\;X_{4}$	none
Misspecification	E $\left(Y|Z,\;X\right)=\alpha +\beta Z+\gamma_{3}X_{3}+\gamma_{5}X_{5}$	$X_{3}$	$X_{8}$
All-variables	E(Y|Z, X)=α+βZ+ $\gamma_{1}X_{1}+\gamma_{2}X_{2}+\gamma_{3}X_{3}+\gamma_{4}X_{4}+\gamma_{5}X_{5}+\gamma_{6}X_{6}+\gamma_{7}X_{7}+\gamma_{8}X_{8}$	$X_{1},X_{2},\;X_{3},\;X_{4}$	$X_{5},X_{6},\;X_{7},\;X_{8}$

Frequentist estimators were compared on all the three scenarios. Bayesian estimators were compared on all-variables scenario, only.

**Table 2:** Results of the simulation study. In semiparametric locally efficient (SLE)/all-variables scenario, a model selection process based on backward (SLE backward) and forward (SLE forward) stepwise techniques were foreseen.

Method	Scenario	BIAS	SE	MC SD	Power	Type II Error	CP	Type I Error
SLE	All variables	−0.010	0.246	0.015	0.947	0.053	0.941	0.061
SLE Backward	All variables	−0.011	0.247	0.015	0.947	0.053	0.941	0.061
SLE Forward	All variables	−0.010	0.246	0.015	0.947	0.053	0.941	0.061
TMLE	All variables	−0.011	0.248	0.015	0.947	0.053	0.939	0.065
G-Comp	All variables	−0.011	0.248	0.015	0.943	0.057	0.944	0.060
AIPTW	All variables	−0.011	0.248	0.015	0.942	0.058	0.942	0.058
DR	All variables	−0.013	0.298	0.010	0.943	0.057	0.940	0.059
SLE	Mis-specification	−0.010	0.246	0.015	0.856	0.144	0.951	0.051
TMLE	Mis-specification	−0.011	0.248	0.016	0.852	0.148	0.947	0.056
G-Comp	Mis-specification	−0.011	0.248	0.016	0.846	0.154	0.949	0.054
AIPTW	Mis-specification	−0.008	0.244	0.015	0.847	0.153	0.948	0.053
DR	Mis-specification	−0.008	0.244	0.015	0.847	0.153	0.948	0.052
SLE	Correct	−0.008	0.244	0.015	0.949	0.051	0.946	0.056
TMLE	Correct	−0.012	0.296	0.010	0.949	0.051	0.946	0.054
G-Comp	Correct	−0.012	0.296	0.010	0.949	0.051	0.947	0.052
AIPTW	Correct	−0.012	0.298	0.011	0.949	0.051	0.948	0.052
DR	Correct	−0.013	0.295	0.011	0.949	0.051	0.947	0.053
GBCEE	All variables	−0.014	0.147	0.022	0.916	0.084	0.952	0.063
BAC	All variables	−0.015	0.299 ^1^	0.046	0.902	0.098	0.945	0.045
BMA	All variables	−0.051	0.451 ^1^	0.090	0.922	0.078	0.942	0.02

^1^ The value is the standard deviation of the posterior distribution. Semi-parametric Locally Efficient (SLE) Estimator: Targeted Maximum Likelihood Estimator (TMLE); G-Computation (G-Comp); Augmented Inverse Probability Weighting (AIPTW); Doubly Robust (DR); Generalized Bayesian Causal Effect Estimation (GBCEE); Bayesian Adjustment for Confounding (BAC); Bayesian Model Average (BMA).

## Data Availability

The data and code for the analysis are available upon request to the authors.

## References

[1] Ciolino J.D., Palac H.L., Yang A., Vaca M., Belli H.M. (2019). Ideal vs. Real: A Systematic Review on Handling Covariates in Randomized Controlled Trials. BMC Med. Res. Methodol..

[2] Robinson L.D., Jewell N.P. (1991). Some Surprising Results about Covariate Adjustment in Logistic Regression Models. Int. Stat. Rev. Rev. Int. Stat..

[3] Kahan B.C., Jairath V., Doré C.J., Morris T.P. (2014). The Risks and Rewards of Covariate Adjustment in Randomized Trials: An Assessment of 12 Outcomes from 8 Studies. Trials.

[4] Raab G.M., Day S., Sales J. (2000). How to Select Covariates to Include in the Analysis of a Clinical Trial. Control. Clin. Trials.

[5] (2015). Committee for Medicinal Products for Human Use (CHMP) Guideline on Adjustment for Baseline Covariates in Clinical Trials. www.Ema.Europa.Eu/Contact.

[6] Tsiatis A.A. (2004). Locally Efficient Semiparametric Estimators for Functional Measurement Error Models. Biometrika.

[7] Funk M.J., Westreich D., Wiesen C., Stürmer T., Brookhart M.A., Davidian M. (2011). Doubly Robust Estimation of Causal Effects. Am. J. Epidemiol..

[8] Zhang M., Gilbert P.B. (2010). Increasing the Efficiency of Prevention Trials by Incorporating Baseline Covariates. Stat. Commun. Infect. Dis..

[9] Clyde M.A., Ghosh J., Littman M.L. (2011). Bayesian Adaptive Sampling for Variable Selection and Model Averaging. J. Comput. Graph. Stat..

[10] Wang C., Parmigiani G., Dominici F. (2012). Bayesian Effect Estimation Accounting for Adjustment Uncertainty. Biometrics.

[11] Talbot D., Lefebvre G., Atherton J. (2015). The Bayesian Causal Effect Estimation Algorithm. J. Causal Inference.

[12] Ryan E.G., Brock K., Gates S., Slade D. (2020). Do We Need to Adjust for Interim Analyses in a Bayesian Adaptive Trial Design?. BMC Med. Res. Methodol..

[13] Oyama J., Murohara T., Kitakaze M., Ishizu T., Sato Y., Kitagawa K., Kamiya H., Ajioka M., Ishihara M., Dai K. (2016). The Effect of Sitagliptin on Carotid Artery Atherosclerosis in Type 2 Diabetes: The PROLOGUE Randomized Controlled Trial. PLoS Med..

[14] Zhang M., Tsiatis A.A., Davidian M. (2008). Improving Efficiency of Inferences in Randomized Clinical Trials Using Auxiliary Covariates. Biometrics.

[15] Wang A., Nianogo R.A., Arah O.A. (2017). G-Computation of Average Treatment Effects on the Treated and the Untreated. BMC Med. Res. Methodol..

[16] Robins J.M., Rotnitzky A., Zhao L.P. (1994). Estimation of Regression Coefficients When Some Regressors Are Not Always Observed. J. Am. Stat. Assoc..

[17] Laan M.V.D., Rose S. (2011). Targeted Learning: Causal Inference for Observational and Experimental Data.

[18] Glynn A.N., Quinn K.M. (2010). An Introduction to the Augmented Inverse Propensity Weighted Estimator. Polit. Anal..

[19] Liang F., Paulo R., Molina G., Clyde M.A., Berger J.O. (2008). Mixtures of g Priors for Bayesian Variable Selection. J. Am. Stat. Assoc..

[20] (2019). U.S. Food and Drug Administration Adaptive Designs for Clinical Trials of Drugs and Biologics: Guidance for Industry. https://www.fda.gov/regulatory-information/search-fda-guidance-documents/adaptive-design-clinical-trials-drugs-and-biologics-guidance-industry.

[21] Moher D., Hopewell S., Schulz K.F., Montori V., Gøtzsche P.C., Devereaux P.J., Elbourne D., Egger M., Altman D.G. (2010). CONSORT 2010 Explanation and Elaboration: Updated Guidelines for Reporting Parallel Group Randomised Trials. J. Clin. Epidemiol..

[22] ICH Official Web Site: ICH. https://www.ich.org/.

[23] Martens E.P., Pestman W.R., Klungel O.H. (2007). Conditioning on the Propensity Score Can Result in Biased Estimation of Common Measures of Treatment Effect: A Monte Carlo Study (p n/a) by Peter C. Austin, Paul Grootendorst, Sharon-Lise T. Normand, Geoffrey M. Anderson, Statistics in Medicine. Stat. Med..

[24] Volinsky C.T., Madigan D., Raftery A.E., Kronmal R.A. (1997). Bayesian Model Averaging in Proportional Hazard Models: Assessing the Risk of a Stroke. J. R. Stat. Soc. Ser. C.

[25] Goncalves A., Ray P., Soper B., Stevens J., Coyle L., Sales A.P. (2020). Generation and Evaluation of Synthetic Patient Data. BMC Med. Res. Methodol..

